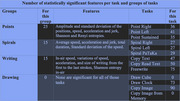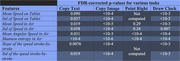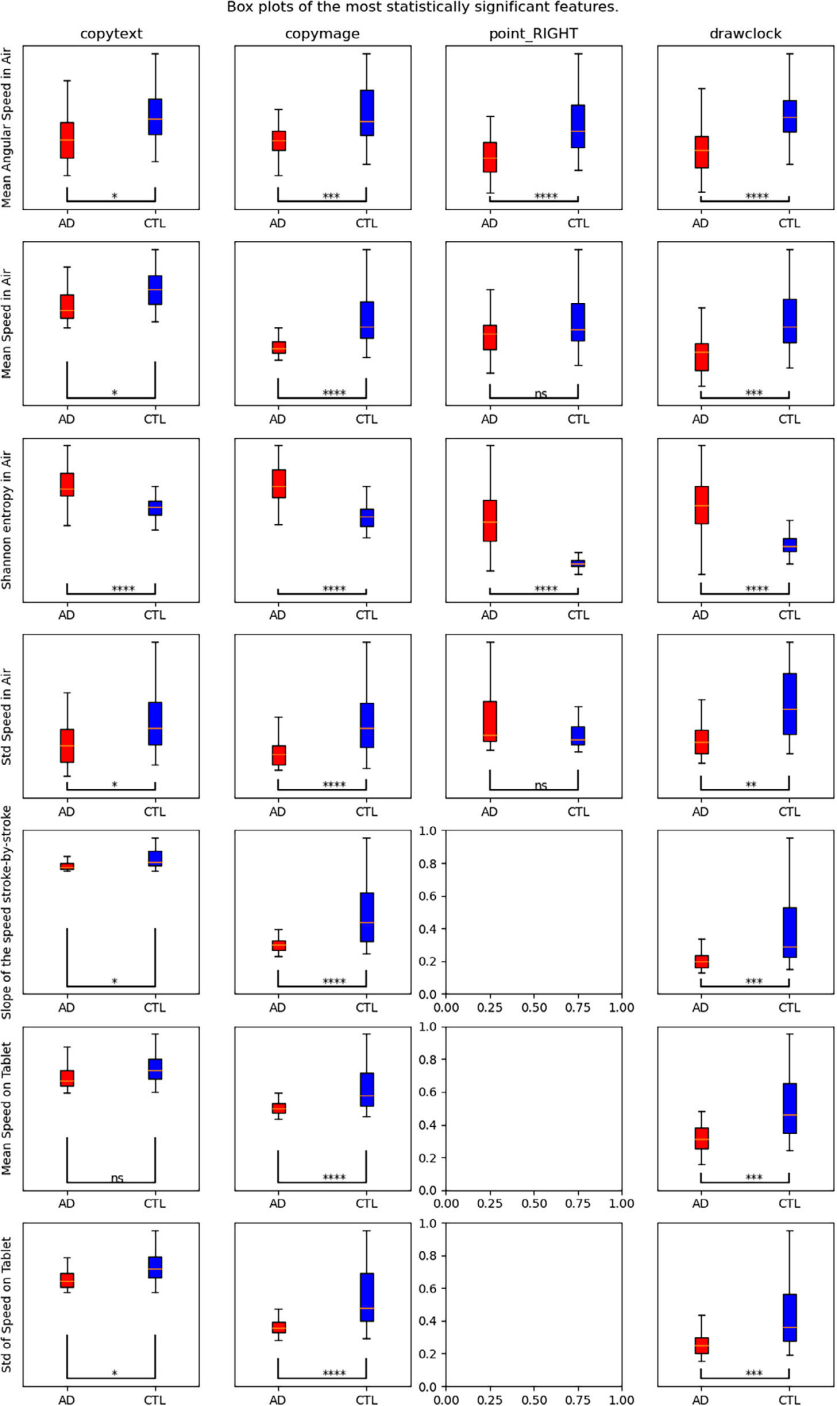# Consistent Explainable features for Alzheimer’s Disease Assessment through Handwriting

**DOI:** 10.1002/alz.092906

**Published:** 2025-01-09

**Authors:** Thomas Thebaud, Esther S Oh, Laureano Moro‐Velazquez, Najim Dehak

**Affiliations:** ^1^ Johns Hopkins University, Baltimore, MD USA; ^2^ Johns Hopkins University School of Medicine, Baltimore, MD USA

## Abstract

**Background:**

Motoric changes are one of the earlier changes in neurodegenerative diseases such as Alzheimer’s disease (AD), but they are often difficult to detect especially in the early stages. In the past, examination of handwriting has been used to investigate motoric changes as a proxy for cognitive impairment. In this study, we explore a wide array of explainable features from the handwriting of patients, obtained using signal processing, and their impact on assessment of AD and Mild cognitive impairment (MCI).

**Method:**

We collected data from 69 participants, 25 with AD or MCI, and 44 for the control group (CTL) on a digital tablet. We analyzed 13 different tasks, categorized into 4 groups: Point tasks (maintaining the pen still, close to the tablet), Spiral tasks (drawing spirals), Writing tasks (paragraphs from different stimuli), and Drawing tasks (drawing various images). Note that not all the participants succeeded to finish all the tasks. From the drawings of each subject were computed multiple characteristics: derivates of the positions, pressions and angles, then we used multiple statistics to get 367 global explainable features.

Those features were compared to obtain the ones providing consistently the most significant differences between the two studied groups using Welch's t‐test, with false discovery rate (FDR) correction. We checked their consistency by comparing which ones were significant across all the tasks of a certain group.

**Result:**

Our results showed that out of the 367 features extracted, 189 features were significantly different between AD/MCI and the control group after FDR‐correction (p‐value<0.05), and 52 were statistically significant for all the tasks of at least one group of tasks.

Most of the significant features were directly related to the velocity with which participants executed the various tasks (average speed, angular speed, variations of the speed on tablet, etc.), and related to the non‐effective movements, measured by the entropies.

**Conclusion:**

The analysis of a large variety of handwriting segments employing machine learning techniques can provide an assessment of patients with AD and MCI that may be useful in their early diagnosis.